# Supplementary material for: The impact of different agroecological conditions on the nutritional composition of quinoa seeds
Source: PeerJ. 2018 Mar 14;6:e4442. doi: 10.7717/peerj.4442 (PMC5857176; doi:10.7717/peerj.4442)
Supplement: Table S1 — Values are Mean ± Stnd Dev (n = 4). [file peerj-06-4442-s001.docx]

| **Variety** | **Location** | **Calcium** | **Iron** | **Magnesium** | **Phosphorus** | **Potassium** | **Sodium** | **Zinc** |
| --- | --- | --- | --- | --- | --- | --- | --- | --- |
| Regalona | Chile | 1265.527±30.239 | 90.983±1.547 | 2278.507±48.31 | 3437.897±135.709 | 13856.503±416.787 | 12.103±0.284 | 40.867±0.342 |
| Salcedo | Chile | 1360.173±27.003 | 83.323±0.503 | 2238.103±39.441 | 3246.087±78.401 | 10006.25±239.102 | 11.383±0.518 | 42.693±0.777 |
| Titicaca | Chile | 618.957±43.568 | 82.54±3.638 | 1813.973±50.908 | 2846.433±79.996 | 10250.257±395.539 | 5.16±0.142 | 40.807±0.764 |
| Regalona | Spain | 728.957±15.75 | 55.383±1.786 | 1962.883±33.73 | 4232.863±108.382 | 11440.323±144.406 | 3.117±0.172 | 25.43±0.082 |
| Salcedo | Spain | 934.45±52.829 | 66.803±2.345 | 1741.167±19.827 | 3155.847±65.912 | 8866.917±174.242 | 16.71±0.948 | 25.267±0.49 |
| Titicaca | Spain | 888.41±92.433 | 69.263±2.028 | 1863.863±54.529 | 3915.373±105.79 | 14678.487±564.942 | 16.747±1.355 | 25.13±0.157 |
| Salcedo | Peru | 513.997±4.311 | 62.81±2.451 | 1924.093±22.15 | 3934.627±11.711 | 9648.66±72.284 | 5.147±0.351 | 32.983±0.148 |

**Supplementary Table 1.** Absolute values of mineral of three cultivars of *C. quinoa* seeds growing at three different locations. Values are Mean ± Stnd Dev (n=4).
