# Supplementary material for: The impact of different agroecological conditions on the nutritional composition of quinoa seeds
Source: PeerJ. 2018 Mar 14;6:e4442. doi: 10.7717/peerj.4442 (PMC5857176; doi:10.7717/peerj.4442)
Supplement: Table S2 — Different letters indicate statistical differences at a P < 0.05 (Duncan t-test). Values represent Mean ± SE. [file peerj-06-4442-s002.docx]

| **Location** | | Regalona | Regalona | Salcedo | Salcedo | Salcedo | Titicaca | Titicaca |
| --- | --- | --- | --- | --- | --- | --- | --- | --- |
| **Variety** | | Chile | Spain | Chile | Peru | Spain | Chile | Spain |
| Amino acid (µg/g) | **Ala)nine** | 52.1±14.6 ab | 49.6±14.6 ab | 35.6±14.6 ab | 33.4±14.6 ab | 108.9±14.6 b | 65±11.9 ab | 26.4±14.6 a |
|  | **Arginine** | 213.6±32.6 ab | 308.4±32.6 b | 130.6±32.6 a | 182.7±32.6 ab | 316±32.6 b | 119.9±26.6 a | 171.8±32.6 ab |
|  | **Asparagine** | 12.1±3.1 a | 20.1±2.5 a | 14.6±3.1 a | 10.4±3.1 a | 22.5±3.1 a | 11.5±2.5 a | 8.6±3.1 a |
|  | **Aspartic acid** | 17.4±5.6 a | 52.9±5.6 bc | 23.2±5.6 ab | 26.3±5.6 ab | 57.7±5.6 c | 19.2±4.6 a | 16.1±5.6 a |
|  | **Glutamic acid** | 288.1±56.1 a | 342.3±45.8 a | 257.1±56.1 a | 175.2±56.1 a | 367.1±56.1 a | 345.7±45.8 a | 181.4±56.1 a |
|  | **Glutamine** | 134.8±21.7 a | 184.9±21.7 a | 164±21.7 a | 118.1±21.7 a | 105.4±21.7 a | 142.4±17.7 a | 117.9±21.7 a |
|  | **Glycine** | 7.9±3.8 a | 15.2±3.1 a | 6.3±3.8 a | 3.7±3.8 a | 16.8±3.8 a | 12.4±3.1 a | 4.6±3.8 a |
|  | **Histidine** | 19.5±2.7 a | 17.8±2.2 a | 11.5±2.7 a | 15.3±2.7 a | 18.8±2.7 a | 16.1±2.2 a | 15.9±2.7 a |
|  | **Hydroxyproline** | 5.1±0.6 a | 4.8±0.5 a | 3.6±0.6 a | 3.6±0.6 a | 4±0.6 a | 3.9±0.5 a | 4.1±0.6 a |
|  | **Isoleucine** | 31.3±4.5 ab | 38.3±3.7 ab | 20.4±4.5 a | 16.9±4.5 a | 51.4±4.5 b | 35.5±3.7 ab | 21.9±4.5 a |
|  | **Leucine** | 35.4±4 ab | 36.2±3.3 ab | 22.2±4 a | 19.4±4 a | 43.6±4 b | 37.5±3.3 ab | 24.3±4 ab |
|  | **Lysine** | 11.9±1 b | 18.7±0.8 c | 7.7±1 ab | 4.1±1 a | 10.5±1 b | 9.1±0.8 b | 8.3±1 ab |
|  | **Methionine** | 4.2±0.9 a | 9.2±0.8 b | 5±0.9 ab | 6.8±0.9 ab | 7.3±0.9 ab | 2.9±0.8 a | 5.6±0.9 ab |
|  | **Phenylalanine** | 25.1±3.4 a | 32.2±2.7 ab | 21±3.4 a | 29.3±3.4 ab | 43.1±3.4 b | 25.2±2.7 a | 19.7±3.4 a |
|  | **Proline** | 87.1±10.2 ab | 87.6±8.3 b | 72.3±10.2 ab | 36.5±10.2 a | 56.4±10.2 ab | 77.4±8.3 ab | 61.5±10.2 ab |
|  | **Serine** | 9.7±2.3 a | 15.9±2.3 a | 12.6±2.3 a | 8.5±2.3 a | 18.4±2.3 a | 10.8±1.9 a | 9.7±2.3 a |
|  | **Threonine** | 30.3±5.6 a | 39.8±4.6 a | 21.1±5.6 a | 17.8±5.6 a | 34.2±5.6 a | 28.8±4.6 a | 17.8±5.6 a |
|  | **Tryptophan** | 34.8±6.2 a | 43.2±5.1 a | 56.6±6.2 a | 121.1±6.2 b | 57.4±6.2 a | 29.2±5.1 a | 32.8±6.2 a |
|  | **Valine** | 31.8±7.9 a | 48.3±6.4 ab | 25.5±7.9 a | 32.7±7.9 a | 75±7.9 b | 35.8±6.4 a | 22.9±7.9 a |

**Supplementary Table 2.** Amino acid content of three *Chenopodium quinoa* cultivars growing under different agroecological conditions. Different letters indicate statistical differences at a *P < 0.05* (Duncan t-test). Values represent Mean ± SE.
