# Supplementary material for: The impact of different agroecological conditions on the nutritional composition of quinoa seeds
Source: PeerJ. 2018 Mar 14;6:e4442. doi: 10.7717/peerj.4442 (PMC5857176; doi:10.7717/peerj.4442)
Supplement: Data S1 — FRAP and protein content were determined as described in the Methods section. Data is presented in Figs. 3 and 5. [file peerj-06-4442-s003.docx]

| SAMPLE | VAR | LOC | VARLOC | N.SAMPLEWEIGHT | H2SO4 0.100 N | PROT.PERCENT | Sample Weight.FRAP | ABS | Curve | uM ET/g | uM ET/100 g | mmol ET/ 100 g |
| --- | --- | --- | --- | --- | --- | --- | --- | --- | --- | --- | --- | --- |
| 1,1 | Salcedo | Spain | Salcedo-Spain | 0,50 | 9,20 | 160,775 | 0,9993 | 0,37 | 307,9 | 308,1 | 30811,57 | 0,31 |
| 1,2 | Salcedo | Spain | Salcedo-Spain | 0,51 | 8,40 | 144,628 | 0,9993 | 0,36 | 296,9 | 297,1 | 29710,8 | 0,3 |
| 2,1 | Regalona | Chile | Regalona-Chile | 0,51 | 10,90 | 188,302 | 10.071 | 0,9 | 838,9 | 833 | 83298,58 | 0,83 |
| 2,2 | Regalona | Chile | Regalona-Chile | 0,51 | 10,00 | 172,857 | 10.071 | 0,82 | 755,9 | 750,6 | 75057,09 | 0,75 |
| 3,1 | Salcedo | Chile | Salcedo-Chile | 0,50 | 9,70 | 170,569 | 0,5066 | 0,31 | 244,9 | 483,4 | 48341,89 | 0,48 |
| 3,2 | Salcedo | Chile | Salcedo-Chile | 0,50 | 9,00 | 156,779 | 0,5066 | 0,28 | 215,9 | 426,2 | 42617,45 | 0,43 |
| 4,1 | Salcedo | Peru | Salcedo-Peru | 0,51 | 8,40 | 144,316 | 10.118 | 0,26 | 192,9 | 190,7 | 19065,03 | 0,19 |
| 4,2 | Salcedo | Peru | Salcedo-Peru | 0,51 | 8,60 | 147,984 | 10.118 | 0,24 | 172,9 | 170,9 | 17088,36 | 0,17 |
| 5,1 | Regalona | Spain | Regalona-Spain | 0,51 | 9,00 | 155,448 | 10.115 | 0,37 | 307,9 | 304,4 | 30439,94 | 0,3 |
| 5,2 | Regalona | Spain | Regalona-Spain | 0,51 | 9,20 | 158,309 | 10.115 | 0,36 | 295,9 | 292,5 | 29253,58 | 0,29 |
| 6,1 | Titicaca | Spain | Titicaca-Spain | 0,50 | 8,70 | 150,892 | 10.054 | 0,77 | 711,9 | 708,1 | 70807,64 | 0,71 |
| 6,2 | Titicaca | Spain | Titicaca-Spain | 0,50 | 8,80 | 153,264 | 10.054 | 0,68 | 615,9 | 612,6 | 61259,2 | 0,61 |
| 7,1 | Titicaca | Chile | Titicaca-Chile | 0,51 | 10,30 | 176,750 | 10.065 | 0,91 | 845,9 | 840,4 | 84043,72 | 0,84 |
| 7,2 | Titicaca | Chile | Titicaca-Chile | 0,51 | 10,40 | 178,747 | 10.065 | 0,8 | 736,9 | 732,1 | 73214,11 | 0,73 |
