# Supplementary material for: The impact of different agroecological conditions on the nutritional composition of quinoa seeds
Source: PeerJ. 2018 Mar 14;6:e4442. doi: 10.7717/peerj.4442 (PMC5857176; doi:10.7717/peerj.4442)
Supplement: Data S2 — Free amino acids were extracted from quinoa Seeds and analyze according to what is described in the Methods section. Data is presented in Fig. 4. [file peerj-06-4442-s004.docx]

| SAMPLE | VAR | LOC | VARLOC | AMINO ACID | CONCENTRATION (µg/g) |
| --- | --- | --- | --- | --- | --- |
| 1 | Salcedo | Spain | Salcedo-Spain | Aspartic acid | 48,230 |
| 15 | Salcedo | Spain | Salcedo-Spain | Aspartic acid | 67,136 |
| 2 | Regalona | Spain | Regalona-Spain | Aspartic acid | 60,007 |
| 9 | Regalona | Spain | Regalona-Spain | Aspartic acid | 45,840 |
| 10 | Titicaca | Spain | Titicaca-Spain | Aspartic acid | 10,105 |
| 17 | Titicaca | Spain | Titicaca-Spain | Aspartic acid | 22,115 |
| 4 | Salcedo | Chile | Salcedo-Chile | Aspartic acid | 25,862 |
| 11 | Salcedo | Chile | Salcedo-Chile | Aspartic acid | 20,579 |
| 12 | Regalona | Chile | Regalona-Chile | Aspartic acid | 14,840 |
| 19 | Regalona | Chile | Regalona-Chile | Aspartic acid | 19,993 |
| 6 | Titicaca | Chile | Titicaca-Chile | Aspartic acid | 25,782 |
| 13 | Titicaca | Chile | Titicaca-Chile | Aspartic acid | 12,944 |
| 20 | Titicaca | Chile | Titicaca-Chile | Aspartic acid | 18,895 |
| 7 | Salcedo | Peru | Salcedo-Peru | Aspartic acid | 30,628 |
| 21 | Salcedo | Peru | Salcedo-Peru | Aspartic acid | 21,929 |
| 1 | Salcedo | Spain | Salcedo-Spain | Glutamic acid | 314,993 |
| 15 | Salcedo | Spain | Salcedo-Spain | Glutamic acid | 419,118 |
| 2 | Regalona | Spain | Regalona-Spain | Glutamic acid | 334,564 |
| 9 | Regalona | Spain | Regalona-Spain | Glutamic acid | 255,971 |
| 16 | Regalona | Spain | Regalona-Spain | Glutamic acid | 436,401 |
| 10 | Titicaca | Spain | Titicaca-Spain | Glutamic acid | 97,056 |
| 17 | Titicaca | Spain | Titicaca-Spain | Glutamic acid | 265,760 |
| 4 | Salcedo | Chile | Salcedo-Chile | Glutamic acid | 273,399 |
| 11 | Salcedo | Chile | Salcedo-Chile | Glutamic acid | 240,854 |
| 12 | Regalona | Chile | Regalona-Chile | Glutamic acid | 253,044 |
| 19 | Regalona | Chile | Regalona-Chile | Glutamic acid | 323,244 |
| 6 | Titicaca | Chile | Titicaca-Chile | Glutamic acid | 435,416 |
| 13 | Titicaca | Chile | Titicaca-Chile | Glutamic acid | 249,125 |
| 20 | Titicaca | Chile | Titicaca-Chile | Glutamic acid | 352,635 |
| 7 | Salcedo | Peru | Salcedo-Peru | Glutamic acid | 187,304 |
| 21 | Salcedo | Peru | Salcedo-Peru | Glutamic acid | 163,084 |
| 1 | Salcedo | Spain | Salcedo-Spain | Alanine | 79,449 |
| 15 | Salcedo | Spain | Salcedo-Spain | Alanine | 138,299 |
| 2 | Regalona | Spain | Regalona-Spain | Alanine | 55,945 |
| 9 | Regalona | Spain | Regalona-Spain | Alanine | 43,336 |
| 10 | Titicaca | Spain | Titicaca-Spain | Alanine | 22,206 |
| 17 | Titicaca | Spain | Titicaca-Spain | Alanine | 30,503 |
| 4 | Salcedo | Chile | Salcedo-Chile | Alanine | 42,488 |
| 11 | Salcedo | Chile | Salcedo-Chile | Alanine | 28,773 |
| 12 | Regalona | Chile | Regalona-Chile | Alanine | 47,755 |
| 19 | Regalona | Chile | Regalona-Chile | Alanine | 56,428 |
| 6 | Titicaca | Chile | Titicaca-Chile | Alanine | 91,778 |
| 13 | Titicaca | Chile | Titicaca-Chile | Alanine | 43,334 |
| 20 | Titicaca | Chile | Titicaca-Chile | Alanine | 59,961 |
| 7 | Salcedo | Peru | Salcedo-Peru | Alanine | 43,783 |
| 21 | Salcedo | Peru | Salcedo-Peru | Alanine | 23,034 |
| 1 | Salcedo | Spain | Salcedo-Spain | Arginine | 342,846 |
| 15 | Salcedo | Spain | Salcedo-Spain | Arginine | 289,066 |
| 9 | Regalona | Spain | Regalona-Spain | Arginine | 318,182 |
| 16 | Regalona | Spain | Regalona-Spain | Arginine | 298,531 |
| 10 | Titicaca | Spain | Titicaca-Spain | Arginine | 94,810 |
| 17 | Titicaca | Spain | Titicaca-Spain | Arginine | 248,793 |
| 4 | Salcedo | Chile | Salcedo-Chile | Arginine | 117,488 |
| 11 | Salcedo | Chile | Salcedo-Chile | Arginine | 143,674 |
| 12 | Regalona | Chile | Regalona-Chile | Arginine | 190,830 |
| 19 | Regalona | Chile | Regalona-Chile | Arginine | 236,360 |
| 6 | Titicaca | Chile | Titicaca-Chile | Arginine | 144,305 |
| 13 | Titicaca | Chile | Titicaca-Chile | Arginine | 86,293 |
| 20 | Titicaca | Chile | Titicaca-Chile | Arginine | 128,985 |
| 7 | Salcedo | Peru | Salcedo-Peru | Arginine | 169,830 |
| 21 | Salcedo | Peru | Salcedo-Peru | Arginine | 195,516 |
| 1 | Salcedo | Spain | Salcedo-Spain | Asparagine | 18,881 |
| 15 | Salcedo | Spain | Salcedo-Spain | Asparagine | 26,087 |
| 2 | Regalona | Spain | Regalona-Spain | Asparagine | 18,648 |
| 9 | Regalona | Spain | Regalona-Spain | Asparagine | 14,831 |
| 16 | Regalona | Spain | Regalona-Spain | Asparagine | 26,745 |
| 10 | Titicaca | Spain | Titicaca-Spain | Asparagine | 6,238 |
| 17 | Titicaca | Spain | Titicaca-Spain | Asparagine | 10,867 |
| 4 | Salcedo | Chile | Salcedo-Chile | Asparagine | 18,288 |
| 11 | Salcedo | Chile | Salcedo-Chile | Asparagine | 10,861 |
| 12 | Regalona | Chile | Regalona-Chile | Asparagine | 10,654 |
| 19 | Regalona | Chile | Regalona-Chile | Asparagine | 13,453 |
| 6 | Titicaca | Chile | Titicaca-Chile | Asparagine | 15,585 |
| 13 | Titicaca | Chile | Titicaca-Chile | Asparagine | 8,067 |
| 20 | Titicaca | Chile | Titicaca-Chile | Asparagine | 10,797 |
| 7 | Salcedo | Peru | Salcedo-Peru | Asparagine | 11,584 |
| 21 | Salcedo | Peru | Salcedo-Peru | Asparagine | 9,214 |
| 1 | Salcedo | Spain | Salcedo-Spain | Phenylalanine | 43,183 |
| 15 | Salcedo | Spain | Salcedo-Spain | Phenylalanine | 42,967 |
| 2 | Regalona | Spain | Regalona-Spain | Phenylalanine | 33,604 |
| 9 | Regalona | Spain | Regalona-Spain | Phenylalanine | 29,854 |
| 16 | Regalona | Spain | Regalona-Spain | Phenylalanine | 33,149 |
| 10 | Titicaca | Spain | Titicaca-Spain | Phenylalanine | 12,974 |
| 17 | Titicaca | Spain | Titicaca-Spain | Phenylalanine | 26,500 |
| 4 | Salcedo | Chile | Salcedo-Chile | Phenylalanine | 20,690 |
| 11 | Salcedo | Chile | Salcedo-Chile | Phenylalanine | 21,341 |
| 12 | Regalona | Chile | Regalona-Chile | Phenylalanine | 22,260 |
| 19 | Regalona | Chile | Regalona-Chile | Phenylalanine | 27,840 |
| 6 | Titicaca | Chile | Titicaca-Chile | Phenylalanine | 31,170 |
| 13 | Titicaca | Chile | Titicaca-Chile | Phenylalanine | 18,759 |
| 20 | Titicaca | Chile | Titicaca-Chile | Phenylalanine | 25,643 |
| 7 | Salcedo | Peru | Salcedo-Peru | Phenylalanine | 31,414 |
| 21 | Salcedo | Peru | Salcedo-Peru | Phenylalanine | 27,088 |
| 1 | Salcedo | Spain | Salcedo-Spain | Glycine | 11,403 |
| 15 | Salcedo | Spain | Salcedo-Spain | Glycine | 22,251 |
| 2 | Regalona | Spain | Regalona-Spain | Glycine | 11,448 |
| 9 | Regalona | Spain | Regalona-Spain | Glycine | 9,438 |
| 16 | Regalona | Spain | Regalona-Spain | Glycine | 24,862 |
| 10 | Titicaca | Spain | Titicaca-Spain | Glycine | 3,867 |
| 17 | Titicaca | Spain | Titicaca-Spain | Glycine | 5,338 |
| 4 | Salcedo | Chile | Salcedo-Chile | Glycine | 7,389 |
| 11 | Salcedo | Chile | Salcedo-Chile | Glycine | 5,145 |
| 12 | Regalona | Chile | Regalona-Chile | Glycine | 7,230 |
| 19 | Regalona | Chile | Regalona-Chile | Glycine | 8,595 |
| 6 | Titicaca | Chile | Titicaca-Chile | Glycine | 18,471 |
| 13 | Titicaca | Chile | Titicaca-Chile | Glycine | 7,879 |
| 20 | Titicaca | Chile | Titicaca-Chile | Glycine | 10,990 |
| 7 | Salcedo | Peru | Salcedo-Peru | Glycine | 4,908 |
| 21 | Salcedo | Peru | Salcedo-Peru | Glycine | 2,580 |
| 1 | Salcedo | Spain | Salcedo-Spain | Glutamine | 93,283 |
| 15 | Salcedo | Spain | Salcedo-Spain | Glutamine | 117,583 |
| 2 | Regalona | Spain | Regalona-Spain | Glutamine | 200,702 |
| 9 | Regalona | Spain | Regalona-Spain | Glutamine | 169,106 |
| 10 | Titicaca | Spain | Titicaca-Spain | Glutamine | 86,577 |
| 17 | Titicaca | Spain | Titicaca-Spain | Glutamine | 149,276 |
| 4 | Salcedo | Chile | Salcedo-Chile | Glutamine | 174,384 |
| 11 | Salcedo | Chile | Salcedo-Chile | Glutamine | 153,582 |
| 12 | Regalona | Chile | Regalona-Chile | Glutamine | 124,619 |
| 19 | Regalona | Chile | Regalona-Chile | Glutamine | 144,993 |
| 6 | Titicaca | Chile | Titicaca-Chile | Glutamine | 189,713 |
| 13 | Titicaca | Chile | Titicaca-Chile | Glutamine | 102,801 |
| 20 | Titicaca | Chile | Titicaca-Chile | Glutamine | 134,769 |
| 7 | Salcedo | Peru | Salcedo-Peru | Glutamine | 133,704 |
| 21 | Salcedo | Peru | Salcedo-Peru | Glutamine | 102,457 |
| 1 | Salcedo | Spain | Salcedo-Spain | Histidine | 21,311 |
| 15 | Salcedo | Spain | Salcedo-Spain | Histidine | 16,304 |
| 2 | Regalona | Spain | Regalona-Spain | Histidine | 19,202 |
| 9 | Regalona | Spain | Regalona-Spain | Histidine | 18,297 |
| 16 | Regalona | Spain | Regalona-Spain | Histidine | 15,821 |
| 10 | Titicaca | Spain | Titicaca-Spain | Histidine | 9,855 |
| 17 | Titicaca | Spain | Titicaca-Spain | Histidine | 21,924 |
| 4 | Salcedo | Chile | Salcedo-Chile | Histidine | 10,345 |
| 11 | Salcedo | Chile | Salcedo-Chile | Histidine | 12,576 |
| 12 | Regalona | Chile | Regalona-Chile | Histidine | 16,743 |
| 19 | Regalona | Chile | Regalona-Chile | Histidine | 22,235 |
| 6 | Titicaca | Chile | Titicaca-Chile | Histidine | 18,663 |
| 13 | Titicaca | Chile | Titicaca-Chile | Histidine | 12,194 |
| 20 | Titicaca | Chile | Titicaca-Chile | Histidine | 17,545 |
| 7 | Salcedo | Peru | Salcedo-Peru | Histidine | 15,314 |
| 21 | Salcedo | Peru | Salcedo-Peru | Histidine | 15,295 |
| 1 | Salcedo | Spain | Salcedo-Spain | Isoleucine | 49,165 |
| 15 | Salcedo | Spain | Salcedo-Spain | Isoleucine | 53,708 |
| 2 | Regalona | Spain | Regalona-Spain | Isoleucine | 37,851 |
| 9 | Regalona | Spain | Regalona-Spain | Isoleucine | 33,513 |
| 16 | Regalona | Spain | Regalona-Spain | Isoleucine | 43,508 |
| 10 | Titicaca | Spain | Titicaca-Spain | Isoleucine | 14,097 |
| 17 | Titicaca | Spain | Titicaca-Spain | Isoleucine | 29,741 |
| 4 | Salcedo | Chile | Salcedo-Chile | Isoleucine | 20,874 |
| 11 | Salcedo | Chile | Salcedo-Chile | Isoleucine | 20,008 |
| 12 | Regalona | Chile | Regalona-Chile | Isoleucine | 29,110 |
| 19 | Regalona | Chile | Regalona-Chile | Isoleucine | 33,445 |
| 6 | Titicaca | Chile | Titicaca-Chile | Isoleucine | 44,253 |
| 13 | Titicaca | Chile | Titicaca-Chile | Isoleucine | 26,451 |
| 20 | Titicaca | Chile | Titicaca-Chile | Isoleucine | 35,861 |
| 7 | Salcedo | Peru | Salcedo-Peru | Isoleucine | 20,026 |
| 21 | Salcedo | Peru | Salcedo-Peru | Isoleucine | 13,821 |
| 1 | Salcedo | Spain | Salcedo-Spain | Leucine | 46,174 |
| 15 | Salcedo | Spain | Salcedo-Spain | Leucine | 41,049 |
| 2 | Regalona | Spain | Regalona-Spain | Leucine | 35,820 |
| 9 | Regalona | Spain | Regalona-Spain | Leucine | 35,439 |
| 16 | Regalona | Spain | Regalona-Spain | Leucine | 37,293 |
| 10 | Titicaca | Spain | Titicaca-Spain | Leucine | 16,093 |
| 17 | Titicaca | Spain | Titicaca-Spain | Leucine | 32,600 |
| 4 | Salcedo | Chile | Salcedo-Chile | Leucine | 20,690 |
| 11 | Salcedo | Chile | Salcedo-Chile | Leucine | 23,628 |
| 12 | Regalona | Chile | Regalona-Chile | Leucine | 33,105 |
| 19 | Regalona | Chile | Regalona-Chile | Leucine | 37,743 |
| 6 | Titicaca | Chile | Titicaca-Chile | Leucine | 43,869 |
| 13 | Titicaca | Chile | Titicaca-Chile | Leucine | 29,640 |
| 20 | Titicaca | Chile | Titicaca-Chile | Leucine | 39,139 |
| 7 | Salcedo | Peru | Salcedo-Peru | Leucine | 22,775 |
| 21 | Salcedo | Peru | Salcedo-Peru | Leucine | 16,032 |
| 1 | Salcedo | Spain | Salcedo-Spain | Lysine | 10,282 |
| 15 | Salcedo | Spain | Salcedo-Spain | Lysine | 10,742 |
| 2 | Regalona | Spain | Regalona-Spain | Lysine | 18,464 |
| 9 | Regalona | Spain | Regalona-Spain | Lysine | 17,334 |
| 16 | Regalona | Spain | Regalona-Spain | Lysine | 20,342 |
| 10 | Titicaca | Spain | Titicaca-Spain | Lysine | 7,485 |
| 17 | Titicaca | Spain | Titicaca-Spain | Lysine | 9,151 |
| 4 | Salcedo | Chile | Salcedo-Chile | Lysine | 7,389 |
| 11 | Salcedo | Chile | Salcedo-Chile | Lysine | 8,003 |
| 12 | Regalona | Chile | Regalona-Chile | Lysine | 10,654 |
| 19 | Regalona | Chile | Regalona-Chile | Lysine | 13,079 |
| 6 | Titicaca | Chile | Titicaca-Chile | Lysine | 10,390 |
| 13 | Titicaca | Chile | Titicaca-Chile | Lysine | 6,941 |
| 20 | Titicaca | Chile | Titicaca-Chile | Lysine | 9,833 |
| 7 | Salcedo | Peru | Salcedo-Peru | Lysine | 5,301 |
| 21 | Salcedo | Peru | Salcedo-Peru | Lysine | 2,948 |
| 1 | Salcedo | Spain | Salcedo-Spain | Methionine | 6,730 |
| 15 | Salcedo | Spain | Salcedo-Spain | Methionine | 7,864 |
| 2 | Regalona | Spain | Regalona-Spain | Methionine | 8,863 |
| 9 | Regalona | Spain | Regalona-Spain | Methionine | 7,897 |
| 16 | Regalona | Spain | Regalona-Spain | Methionine | 10,736 |
| 10 | Titicaca | Spain | Titicaca-Spain | Methionine | 3,618 |
| 17 | Titicaca | Spain | Titicaca-Spain | Methionine | 7,626 |
| 4 | Salcedo | Chile | Salcedo-Chile | Methionine | 4,988 |
| 11 | Salcedo | Chile | Salcedo-Chile | Methionine | 4,954 |
| 12 | Regalona | Chile | Regalona-Chile | Methionine | 3,805 |
| 19 | Regalona | Chile | Regalona-Chile | Methionine | 4,671 |
| 6 | Titicaca | Chile | Titicaca-Chile | Methionine | 2,309 |
| 13 | Titicaca | Chile | Titicaca-Chile | Methionine | 2,814 |
| 20 | Titicaca | Chile | Titicaca-Chile | Methionine | 3,470 |
| 7 | Salcedo | Peru | Salcedo-Peru | Methionine | 7,853 |
| 21 | Salcedo | Peru | Salcedo-Peru | Methionine | 5,713 |
| 1 | Salcedo | Spain | Salcedo-Spain | Proline | 57,764 |
| 15 | Salcedo | Spain | Salcedo-Spain | Proline | 55,051 |
| 2 | Regalona | Spain | Regalona-Spain | Proline | 88,996 |
| 9 | Regalona | Spain | Regalona-Spain | Proline | 78,968 |
| 16 | Regalona | Spain | Regalona-Spain | Proline | 94,927 |
| 10 | Titicaca | Spain | Titicaca-Spain | Proline | 44,910 |
| 17 | Titicaca | Spain | Titicaca-Spain | Proline | 78,165 |
| 4 | Salcedo | Chile | Salcedo-Chile | Proline | 73,522 |
| 11 | Salcedo | Chile | Salcedo-Chile | Proline | 71,075 |
| 12 | Regalona | Chile | Regalona-Chile | Proline | 72,298 |
| 19 | Regalona | Chile | Regalona-Chile | Proline | 101,831 |
| 6 | Titicaca | Chile | Titicaca-Chile | Proline | 91,393 |
| 13 | Titicaca | Chile | Titicaca-Chile | Proline | 55,715 |
| 20 | Titicaca | Chile | Titicaca-Chile | Proline | 85,219 |
| 7 | Salcedo | Peru | Salcedo-Peru | Proline | 37,500 |
| 21 | Salcedo | Peru | Salcedo-Peru | Proline | 35,565 |
| 1 | Salcedo | Spain | Salcedo-Spain | Serine | 14,768 |
| 15 | Salcedo | Spain | Salcedo-Spain | Serine | 22,059 |
| 2 | Regalona | Spain | Regalona-Spain | Serine | 17,541 |
| 9 | Regalona | Spain | Regalona-Spain | Serine | 14,253 |
| 10 | Titicaca | Spain | Titicaca-Spain | Serine | 7,859 |
| 17 | Titicaca | Spain | Titicaca-Spain | Serine | 11,629 |
| 4 | Salcedo | Chile | Salcedo-Chile | Serine | 14,409 |
| 11 | Salcedo | Chile | Salcedo-Chile | Serine | 10,861 |
| 12 | Regalona | Chile | Regalona-Chile | Serine | 8,752 |
| 19 | Regalona | Chile | Regalona-Chile | Serine | 10,650 |
| 6 | Titicaca | Chile | Titicaca-Chile | Serine | 15,008 |
| 13 | Titicaca | Chile | Titicaca-Chile | Serine | 7,316 |
| 20 | Titicaca | Chile | Titicaca-Chile | Serine | 10,219 |
| 7 | Salcedo | Peru | Salcedo-Peru | Serine | 10,798 |
| 21 | Salcedo | Peru | Salcedo-Peru | Serine | 6,265 |
| 1 | Salcedo | Spain | Salcedo-Spain | Hydroxyproline | 4,487 |
| 15 | Salcedo | Spain | Salcedo-Spain | Hydroxyproline | 3,453 |
| 2 | Regalona | Spain | Regalona-Spain | Hydroxyproline | 5,170 |
| 9 | Regalona | Spain | Regalona-Spain | Hydroxyproline | 5,008 |
| 16 | Regalona | Spain | Regalona-Spain | Hydroxyproline | 4,144 |
| 10 | Titicaca | Spain | Titicaca-Spain | Hydroxyproline | 2,869 |
| 17 | Titicaca | Spain | Titicaca-Spain | Hydroxyproline | 5,338 |
| 4 | Salcedo | Chile | Salcedo-Chile | Hydroxyproline | 3,140 |
| 11 | Salcedo | Chile | Salcedo-Chile | Hydroxyproline | 4,002 |
| 12 | Regalona | Chile | Regalona-Chile | Hydroxyproline | 4,566 |
| 19 | Regalona | Chile | Regalona-Chile | Hydroxyproline | 5,605 |
| 6 | Titicaca | Chile | Titicaca-Chile | Hydroxyproline | 4,041 |
| 13 | Titicaca | Chile | Titicaca-Chile | Hydroxyproline | 3,377 |
| 20 | Titicaca | Chile | Titicaca-Chile | Hydroxyproline | 4,242 |
| 7 | Salcedo | Peru | Salcedo-Peru | Hydroxyproline | 3,534 |
| 21 | Salcedo | Peru | Salcedo-Peru | Hydroxyproline | 3,686 |
| 1 | Salcedo | Spain | Salcedo-Spain | Threonine | 28,228 |
| 15 | Salcedo | Spain | Salcedo-Spain | Threonine | 40,090 |
| 2 | Regalona | Spain | Regalona-Spain | Threonine | 36,189 |
| 9 | Regalona | Spain | Regalona-Spain | Threonine | 32,165 |
| 16 | Regalona | Spain | Regalona-Spain | Threonine | 51,042 |
| 10 | Titicaca | Spain | Titicaca-Spain | Threonine | 14,097 |
| 17 | Titicaca | Spain | Titicaca-Spain | Threonine | 21,543 |
| 4 | Salcedo | Chile | Salcedo-Chile | Threonine | 23,461 |
| 11 | Salcedo | Chile | Salcedo-Chile | Threonine | 18,674 |
| 12 | Regalona | Chile | Regalona-Chile | Threonine | 27,588 |
| 19 | Regalona | Chile | Regalona-Chile | Threonine | 33,072 |
| 6 | Titicaca | Chile | Titicaca-Chile | Threonine | 39,828 |
| 13 | Titicaca | Chile | Titicaca-Chile | Threonine | 19,697 |
| 20 | Titicaca | Chile | Titicaca-Chile | Threonine | 26,799 |
| 7 | Salcedo | Peru | Salcedo-Peru | Threonine | 21,793 |
| 21 | Salcedo | Peru | Salcedo-Peru | Threonine | 13,821 |
| 1 | Salcedo | Spain | Salcedo-Spain | Tryptophan | 55,147 |
| 15 | Salcedo | Spain | Salcedo-Spain | Tryptophan | 59,655 |
| 2 | Regalona | Spain | Regalona-Spain | Tryptophan | 43,390 |
| 9 | Regalona | Spain | Regalona-Spain | Tryptophan | 37,750 |
| 16 | Regalona | Spain | Regalona-Spain | Tryptophan | 48,594 |
| 10 | Titicaca | Spain | Titicaca-Spain | Tryptophan | 18,588 |
| 17 | Titicaca | Spain | Titicaca-Spain | Tryptophan | 47,089 |
| 4 | Salcedo | Chile | Salcedo-Chile | Tryptophan | 57,081 |
| 11 | Salcedo | Chile | Salcedo-Chile | Tryptophan | 56,212 |
| 12 | Regalona | Chile | Regalona-Chile | Tryptophan | 29,680 |
| 19 | Regalona | Chile | Regalona-Chile | Tryptophan | 39,985 |
| 6 | Titicaca | Chile | Titicaca-Chile | Tryptophan | 37,327 |
| 13 | Titicaca | Chile | Titicaca-Chile | Tryptophan | 20,073 |
| 20 | Titicaca | Chile | Titicaca-Chile | Tryptophan | 30,270 |
| 7 | Salcedo | Peru | Salcedo-Peru | Tryptophan | 124,084 |
| 21 | Salcedo | Peru | Salcedo-Peru | Tryptophan | 118,120 |
| 1 | Salcedo | Spain | Salcedo-Spain | Valine | 62,625 |
| 15 | Salcedo | Spain | Salcedo-Spain | Valine | 87,468 |
| 2 | Regalona | Spain | Regalona-Spain | Valine | 42,467 |
| 9 | Regalona | Spain | Regalona-Spain | Valine | 38,136 |
| 16 | Regalona | Spain | Regalona-Spain | Valine | 64,227 |
| 10 | Titicaca | Spain | Titicaca-Spain | Valine | 16,093 |
| 17 | Titicaca | Spain | Titicaca-Spain | Valine | 29,741 |
| 4 | Salcedo | Chile | Salcedo-Chile | Valine | 26,970 |
| 11 | Salcedo | Chile | Salcedo-Chile | Valine | 24,009 |
| 12 | Regalona | Chile | Regalona-Chile | Valine | 28,919 |
| 19 | Regalona | Chile | Regalona-Chile | Valine | 34,753 |
| 6 | Titicaca | Chile | Titicaca-Chile | Valine | 46,755 |
| 13 | Titicaca | Chile | Titicaca-Chile | Valine | 26,076 |
| 20 | Titicaca | Chile | Titicaca-Chile | Valine | 34,512 |
| 7 | Salcedo | Peru | Salcedo-Peru | Valine | 39,071 |
| 21 | Salcedo | Peru | Salcedo-Peru | Valine | 26,351 |
