# Supplementary material for: The impact of different agroecological conditions on the nutritional composition of quinoa seeds
Source: PeerJ. 2018 Mar 14;6:e4442. doi: 10.7717/peerj.4442 (PMC5857176; doi:10.7717/peerj.4442)
Supplement: Data S3 — Agronomical parameters presented in Table 1 were determined in the three quinoa cultivars at the three locations. [file peerj-06-4442-s005.docx]

| LOC | Variety | Sample | Yield (kg/ha) | Days to flowering | Days to maturity | Plant Height (m) | Shoot diameter (mm) | Panicle lenght (cm) | Panicle diameter (cm) | Plant weight (g) | Seed weight per plant (g) | Harvest Index |
| --- | --- | --- | --- | --- | --- | --- | --- | --- | --- | --- | --- | --- |
| España | Salcedo | SCD1 | (*) | 92 | 187 | 1,28 | 13 | 35 | 8 | (*) | (*) | (*) |
| España | Salcedo | SCD2 | (*) | 92 | 187 | 1,34 | 16 | 34 | 8 | (*) | (*) | (*) |
| España | Salcedo | SCD3 | (*) | 92 | 187 | 1,43 | 20 | 37 | 10 | (*) | (*) | (*) |
| España | Salcedo | SCD4 | (*) | 92 | 187 | 1,41 | 19 | 40 | 10 | (*) | (*) | (*) |
| España | Salcedo | SCD5 | (*) | 92 | 187 | 1,35 | 16 | 34 | 9 | (*) | (*) | (*) |
| España | Titicaca | TIC1 | 1526 | 51 | 119 | 1,18 | 13 | 32 | 8 | 98 | 41 | 0,42 |
| España | Titicaca | TIC2 | 1526 | 51 | 119 | 1,14 | 12 | 27 | 7 | 90 | 38 | 0,42 |
| España | Titicaca | TIC3 | 1526 | 51 | 119 | 1,19 | 14 | 28 | 8 | 96 | 42 | 0,44 |
| España | Titicaca | TIC4 | 1526 | 51 | 119 | 1,12 | 11 | 27 | 6 | 84 | 31 | 0,37 |
| España | Titicaca | TIC5 | 1526 | 51 | 119 | 1,13 | 11 | 26 | 6 | 80 | 34 | 0,43 |
| España | Regalona | REG1 | 2606 | 63 | 138 | 1,19 | 12 | 17 | 10 | 89 | 38 | 0,43 |
| España | Regalona | REG2 | 2606 | 63 | 138 | 1,21 | 14 | 20 | 14 | 117 | 62 | 0,53 |
| España | Regalona | REG3 | 2606 | 63 | 138 | 1,32 | 18 | 24 | 13 | 128 | 58 | 0,45 |
| España | Regalona | REG4 | 2606 | 63 | 138 | 1,27 | 16 | 20 | 12 | 101 | 46 | 0,46 |
| España | Regalona | REG5 | 2606 | 63 | 138 | 1,24 | 14 | 19 | 11 | 104 | 49 | 0,47 |
| Chile | Salcedo | 3 | 2714 | 100 | 180 | 0,85 | 10 | 35 | 7 | 77 | 44 | 0,57 |
| Chile | Salcedo | 3 | 2682 | 100 | 180 | 0,75 | 11 | 32 | 7 | 76 | 30 | 0,4 |
| Chile | Salcedo | 3 | 2834 | 100 | 180 | 0,92 | 13 | 28 | 7 | 81 | 36 | 0,44 |
| Chile | Titicaca | 7 | 2270 | 50 | 105 | 1 | 13 | 23 | 7 | 65 | 32 | 0,5 |
| Chile | Titicaca | 7 | 5863 | 50 | 105 | 1,14 | 11 | 20 | 7 | 103 | 51 | 0,5 |
| Chile | Titicaca | 7 | 4768 | 50 | 105 | 0,98 | 12 | 24 | 7 | 90 | 45 | 0,5 |
| Chile | Regalona | 9 | 2939 | 70 | 165 | 1,25 | 17 | 18 | 11 | 84 | 56 | 0,67 |
| Chile | Regalona | 9 | 2156 | 70 | 165 | 1,29 | 14 | 17 | 10 | 105 | 35 | 0,33 |
| Chile | Regalona | 9 | 2112 | 70 | 165 | 1,36 | 15 | 16 | 12 | 120 | 30 | 0,25 |
| Peru | Salcedo | SCD1 | 5160 | 66 | 146 | 1,34 | 19 | 34 | 16 | 65 | 26 | 0,4 |
| Peru | Salcedo | SCD2 | 5320 | 65 | 145 | 1,37 | 18 | 35 | 15 | 67 | 27 | 0,4 |
| Peru | Salcedo | SCD3 | 4980 | 67 | 144 | 1,36 | 18 | 37 | 15 | 63 | 25 | 0,4 |
| Peru | Salcedo | SCD4 | 5220 | 62 | 145 | 1,37 | 18 | 36 | 16 | 69 | 28 | 0,4 |
