# Supplementary material for: The impact of different agroecological conditions on the nutritional composition of quinoa seeds
Source: PeerJ. 2018 Mar 14;6:e4442. doi: 10.7717/peerj.4442 (PMC5857176; doi:10.7717/peerj.4442)
Supplement: Data S4 — Fiber and Saponin were determined in quinoa seeds as described in the Methods section. Data is presented in figures 6 and 7. [file peerj-06-4442-s006.docx]

| VARLOC | VAR | LOC | VALUE (%) | VARIABLE |
| --- | --- | --- | --- | --- |
| Titicaca-Spain | Titicaca | Spain | 0,9 | SAPONIN.PERC |
| Titicaca-Spain | Titicaca | Spain | 1,6 | SAPONIN.PERC |
| Titicaca-Chile | Titicaca | Chile | 1,1 | SAPONIN.PERC |
| Titicaca-Chile | Titicaca | Chile | 1,3 | SAPONIN.PERC |
| Regalona-Spain | Regalona | Spain | 1,48 | SAPONIN.PERC |
| Regalona-Spain | Regalona | Spain | 1,34 | SAPONIN.PERC |
| Regalona-Chile | Regalona | Chile | 1,3 | SAPONIN.PERC |
| Regalona-Chile | Regalona | Chile | 1,38 | SAPONIN.PERC |
| Salcedo-Peru | Salcedo | Peru | 0,62 | SAPONIN.PERC |
| Salcedo-Peru | Salcedo | Peru | 1,07 | SAPONIN.PERC |
| Salcedo-Spain | Salcedo | Spain | 0,73 | SAPONIN.PERC |
| Salcedo-Spain | Salcedo | Spain | 1 | SAPONIN.PERC |
| Salcedo-Chile | Salcedo | Chile | 1,1 | SAPONIN.PERC |
| Salcedo-Chile | Salcedo | Chile | 0,8 | SAPONIN.PERC |
| Titicaca-Spain | Titicaca | Spain | 3,4 | FIBER.PERC |
| Titicaca-Spain | Titicaca | Spain | 3,5 | FIBER.PERC |
| Titicaca-Chile | Titicaca | Chile | 3,8 | FIBER.PERC |
| Titicaca-Chile | Titicaca | Chile | 3,4 | FIBER.PERC |
| Regalona-Spain | Regalona | Spain | 2,91 | FIBER.PERC |
| Regalona-Spain | Regalona | Spain | 3,1 | FIBER.PERC |
| Regalona-Chile | Regalona | Chile | 2,95 | FIBER.PERC |
| Regalona-Chile | Regalona | Chile | 3,15 | FIBER.PERC |
| Salcedo-Peru | Salcedo | Peru | 3,2 | FIBER.PERC |
| Salcedo-Peru | Salcedo | Peru | 3,4 | FIBER.PERC |
| Salcedo-Spain | Salcedo | Spain | 2,9 | FIBER.PERC |
| Salcedo-Spain | Salcedo | Spain | 3,4 | FIBER.PERC |
| Salcedo-Chile | Salcedo | Chile | 3,05 | FIBER.PERC |
| Salcedo-Chile | Salcedo | Chile | 3,45 | FIBER.PERC |
