# Supplementary material for: The impact of different agroecological conditions on the nutritional composition of quinoa seeds
Source: PeerJ. 2018 Mar 14;6:e4442. doi: 10.7717/peerj.4442 (PMC5857176; doi:10.7717/peerj.4442)
Supplement: Data S5 — Mineral composition of seeds was determined as described in the Methods section and final data is presented in Fig. 1. [file peerj-06-4442-s007.docx]

| SAMPLE | VAR | LOC | VARLOC | ELEMENT | CONCENTRATION  (µg/g) |
| --- | --- | --- | --- | --- | --- |
| 1 | Salcedo | Spain | Salcedo-Spain | Calcium | 925,48 |
| 1 | Salcedo | Spain | Salcedo-Spain | Iron | 66,84 |
| 1 | Salcedo | Spain | Salcedo-Spain | Magnesium | 1731,31 |
| 1 | Salcedo | Spain | Salcedo-Spain | Phosphorus | 3158,01 |
| 1 | Salcedo | Spain | Salcedo-Spain | Potassium | 8833,66 |
| 1 | Salcedo | Spain | Salcedo-Spain | Sodium | 16,62 |
| 1 | Salcedo | Spain | Salcedo-Spain | Zinc | 25,35 |
| 2 | Regalona | Spain | Regalona-Spain | Calcium | 711,54 |
| 2 | Regalona | Spain | Regalona-Spain | Iron | 57,25 |
| 2 | Regalona | Spain | Regalona-Spain | Magnesium | 1994,36 |
| 2 | Regalona | Spain | Regalona-Spain | Phosphorus | 4298,32 |
| 2 | Regalona | Spain | Regalona-Spain | Potassium | 11321,75 |
| 2 | Regalona | Spain | Regalona-Spain | Sodium | 3,31 |
| 2 | Regalona | Spain | Regalona-Spain | Zinc | 25,41 |
| 3 | Titicaca | Spain | Titicaca-Spain | Calcium | 993,82 |
| 3 | Titicaca | Spain | Titicaca-Spain | Iron | 71,58 |
| 3 | Titicaca | Spain | Titicaca-Spain | Magnesium | 1904,36 |
| 3 | Titicaca | Spain | Titicaca-Spain | Phosphorus | 3897,8 |
| 3 | Titicaca | Spain | Titicaca-Spain | Potassium | 15329,17 |
| 3 | Titicaca | Spain | Titicaca-Spain | Sodium | 18,31 |
| 3 | Titicaca | Spain | Titicaca-Spain | Zinc | 25,06 |
| 4 | Salcedo | Chile | Salcedo-Chile | Calcium | 1356,91 |
| 4 | Salcedo | Chile | Salcedo-Chile | Iron | 83,79 |
| 4 | Salcedo | Chile | Salcedo-Chile | Magnesium | 2193,39 |
| 4 | Salcedo | Chile | Salcedo-Chile | Phosphorus | 3167,42 |
| 4 | Salcedo | Chile | Salcedo-Chile | Potassium | 9858,14 |
| 4 | Salcedo | Chile | Salcedo-Chile | Sodium | 11,79 |
| 4 | Salcedo | Chile | Salcedo-Chile | Zinc | 43,59 |
| 5 | Regalona | Chile | Regalona-Chile | Calcium | 1256,2 |
| 5 | Regalona | Chile | Regalona-Chile | Iron | 90,62 |
| 5 | Regalona | Chile | Regalona-Chile | Magnesium | 2243,19 |
| 5 | Regalona | Chile | Regalona-Chile | Phosphorus | 3330,03 |
| 5 | Regalona | Chile | Regalona-Chile | Potassium | 13566,28 |
| 5 | Regalona | Chile | Regalona-Chile | Sodium | 12,02 |
| 5 | Regalona | Chile | Regalona-Chile | Zinc | 40,64 |
| 6 | Titicaca | Chile | Titicaca-Chile | Calcium | 577,22 |
| 6 | Titicaca | Chile | Titicaca-Chile | Iron | 78,34 |
| 6 | Titicaca | Chile | Titicaca-Chile | Magnesium | 1755,47 |
| 6 | Titicaca | Chile | Titicaca-Chile | Phosphorus | 2754,08 |
| 6 | Titicaca | Chile | Titicaca-Chile | Potassium | 9813,26 |
| 6 | Titicaca | Chile | Titicaca-Chile | Sodium | 5,32 |
| 6 | Titicaca | Chile | Titicaca-Chile | Zinc | 39,93 |
| 7 | Salcedo | Peru | Salcedo-Peru | Calcium | 514,72 |
| 7 | Salcedo | Peru | Salcedo-Peru | Iron | 61,45 |
| 7 | Salcedo | Peru | Salcedo-Peru | Magnesium | 1927,11 |
| 7 | Salcedo | Peru | Salcedo-Peru | Phosphorus | 3948,06 |
| 7 | Salcedo | Peru | Salcedo-Peru | Potassium | 9722,93 |
| 7 | Salcedo | Peru | Salcedo-Peru | Sodium | 5,12 |
| 7 | Salcedo | Peru | Salcedo-Peru | Zinc | 33,11 |
| 8 | Salcedo | Spain | Salcedo-Spain | Calcium | 991,19 |
| 8 | Salcedo | Spain | Salcedo-Spain | Iron | 69,13 |
| 8 | Salcedo | Spain | Salcedo-Spain | Magnesium | 1763,99 |
| 8 | Salcedo | Spain | Salcedo-Spain | Phosphorus | 3220,65 |
| 8 | Salcedo | Spain | Salcedo-Spain | Potassium | 9055,39 |
| 8 | Salcedo | Spain | Salcedo-Spain | Sodium | 17,7 |
| 8 | Salcedo | Spain | Salcedo-Spain | Zinc | 25,71 |
| 9 | Regalona | Spain | Regalona-Spain | Calcium | 742,2 |
| 9 | Regalona | Spain | Regalona-Spain | Iron | 53,69 |
| 9 | Regalona | Spain | Regalona-Spain | Magnesium | 1927,28 |
| 9 | Regalona | Spain | Regalona-Spain | Phosphorus | 4107,76 |
| 9 | Regalona | Spain | Regalona-Spain | Potassium | 11398,08 |
| 9 | Regalona | Spain | Regalona-Spain | Sodium | 3,06 |
| 9 | Regalona | Spain | Regalona-Spain | Zinc | 25,36 |
| 10 | Titicaca | Spain | Titicaca-Spain | Calcium | 821,2 |
| 10 | Titicaca | Spain | Titicaca-Spain | Iron | 67,81 |
| 10 | Titicaca | Spain | Titicaca-Spain | Magnesium | 1885,37 |
| 10 | Titicaca | Spain | Titicaca-Spain | Phosphorus | 4028,85 |
| 10 | Titicaca | Spain | Titicaca-Spain | Potassium | 14393,37 |
| 10 | Titicaca | Spain | Titicaca-Spain | Sodium | 15,9 |
| 10 | Titicaca | Spain | Titicaca-Spain | Zinc | 25,31 |
| 11 | Salcedo | Chile | Salcedo-Chile | Calcium | 1334,95 |
| 11 | Salcedo | Chile | Salcedo-Chile | Iron | 83,39 |
| 11 | Salcedo | Chile | Salcedo-Chile | Magnesium | 2252,97 |
| 11 | Salcedo | Chile | Salcedo-Chile | Phosphorus | 3246,62 |
| 11 | Salcedo | Chile | Salcedo-Chile | Potassium | 9878,52 |
| 11 | Salcedo | Chile | Salcedo-Chile | Sodium | 11,56 |
| 11 | Salcedo | Chile | Salcedo-Chile | Zinc | 42,27 |
| 12 | Regalona | Chile | Regalona-Chile | Calcium | 1241,05 |
| 12 | Regalona | Chile | Regalona-Chile | Iron | 89,65 |
| 12 | Regalona | Chile | Regalona-Chile | Magnesium | 2258,77 |
| 12 | Regalona | Chile | Regalona-Chile | Phosphorus | 3393,39 |
| 12 | Regalona | Chile | Regalona-Chile | Potassium | 13669,14 |
| 12 | Regalona | Chile | Regalona-Chile | Sodium | 12,42 |
| 12 | Regalona | Chile | Regalona-Chile | Zinc | 41,26 |
| 13 | Titicaca | Chile | Titicaca-Chile | Calcium | 664,15 |
| 13 | Titicaca | Chile | Titicaca-Chile | Iron | 84,58 |
| 13 | Titicaca | Chile | Titicaca-Chile | Magnesium | 1838,26 |
| 13 | Titicaca | Chile | Titicaca-Chile | Phosphorus | 2891,02 |
| 13 | Titicaca | Chile | Titicaca-Chile | Potassium | 10583,76 |
| 13 | Titicaca | Chile | Titicaca-Chile | Sodium | 5,05 |
| 13 | Titicaca | Chile | Titicaca-Chile | Zinc | 41,16 |
| 14 | Salcedo | Peru | Salcedo-Peru | Calcium | 517,9 |
| 14 | Salcedo | Peru | Salcedo-Peru | Iron | 61,34 |
| 14 | Salcedo | Peru | Salcedo-Peru | Magnesium | 1900,59 |
| 14 | Salcedo | Peru | Salcedo-Peru | Phosphorus | 3926,57 |
| 14 | Salcedo | Peru | Salcedo-Peru | Potassium | 9644,51 |
| 14 | Salcedo | Peru | Salcedo-Peru | Sodium | 4,81 |
| 14 | Salcedo | Peru | Salcedo-Peru | Zinc | 32,82 |
| 15 | Salcedo | Spain | Salcedo-Spain | Calcium | 886,68 |
| 15 | Salcedo | Spain | Salcedo-Spain | Iron | 64,44 |
| 15 | Salcedo | Spain | Salcedo-Spain | Magnesium | 1728,2 |
| 15 | Salcedo | Spain | Salcedo-Spain | Phosphorus | 3088,88 |
| 15 | Salcedo | Spain | Salcedo-Spain | Potassium | 8711,7 |
| 15 | Salcedo | Spain | Salcedo-Spain | Sodium | 15,81 |
| 15 | Salcedo | Spain | Salcedo-Spain | Zinc | 24,74 |
| 16 | Regalona | Spain | Regalona-Spain | Calcium | 733,13 |
| 16 | Regalona | Spain | Regalona-Spain | Iron | 55,21 |
| 16 | Regalona | Spain | Regalona-Spain | Magnesium | 1967,01 |
| 16 | Regalona | Spain | Regalona-Spain | Phosphorus | 4292,51 |
| 16 | Regalona | Spain | Regalona-Spain | Potassium | 11601,14 |
| 16 | Regalona | Spain | Regalona-Spain | Sodium | 2,98 |
| 16 | Regalona | Spain | Regalona-Spain | Zinc | 25,52 |
| 17 | Titicaca | Spain | Titicaca-Spain | Calcium | 850,21 |
| 17 | Titicaca | Spain | Titicaca-Spain | Iron | 68,4 |
| 17 | Titicaca | Spain | Titicaca-Spain | Magnesium | 1801,86 |
| 17 | Titicaca | Spain | Titicaca-Spain | Phosphorus | 3819,47 |
| 17 | Titicaca | Spain | Titicaca-Spain | Potassium | 14312,92 |
| 17 | Titicaca | Spain | Titicaca-Spain | Sodium | 16,03 |
| 17 | Titicaca | Spain | Titicaca-Spain | Zinc | 25,02 |
| 18 | Salcedo | Chile | Salcedo-Chile | Calcium | 1388,66 |
| 18 | Salcedo | Chile | Salcedo-Chile | Iron | 82,79 |
| 18 | Salcedo | Chile | Salcedo-Chile | Magnesium | 2267,95 |
| 18 | Salcedo | Chile | Salcedo-Chile | Phosphorus | 3324,22 |
| 18 | Salcedo | Chile | Salcedo-Chile | Potassium | 10282,09 |
| 18 | Salcedo | Chile | Salcedo-Chile | Sodium | 10,8 |
| 18 | Salcedo | Chile | Salcedo-Chile | Zinc | 42,22 |
| 19 | Regalona | Chile | Regalona-Chile | Calcium | 1299,33 |
| 19 | Regalona | Chile | Regalona-Chile | Iron | 92,68 |
| 19 | Regalona | Chile | Regalona-Chile | Magnesium | 2333,56 |
| 19 | Regalona | Chile | Regalona-Chile | Phosphorus | 3590,27 |
| 19 | Regalona | Chile | Regalona-Chile | Potassium | 14334,09 |
| 19 | Regalona | Chile | Regalona-Chile | Sodium | 11,87 |
| 19 | Regalona | Chile | Regalona-Chile | Zinc | 40,7 |
| 20 | Titicaca | Chile | Titicaca-Chile | Calcium | 615,5 |
| 20 | Titicaca | Chile | Titicaca-Chile | Iron | 84,7 |
| 20 | Titicaca | Chile | Titicaca-Chile | Magnesium | 1848,19 |
| 20 | Titicaca | Chile | Titicaca-Chile | Phosphorus | 2894,2 |
| 20 | Titicaca | Chile | Titicaca-Chile | Potassium | 10353,75 |
| 20 | Titicaca | Chile | Titicaca-Chile | Sodium | 5,11 |
| 20 | Titicaca | Chile | Titicaca-Chile | Zinc | 41,33 |
| 21 | Salcedo | Peru | Salcedo-Peru | Calcium | 509,37 |
| 21 | Salcedo | Peru | Salcedo-Peru | Iron | 65,64 |
| 21 | Salcedo | Peru | Salcedo-Peru | Magnesium | 1944,58 |
| 21 | Salcedo | Peru | Salcedo-Peru | Phosphorus | 3929,25 |
| 21 | Salcedo | Peru | Salcedo-Peru | Potassium | 9578,54 |
| 21 | Salcedo | Peru | Salcedo-Peru | Sodium | 5,51 |
| 21 | Salcedo | Peru | Salcedo-Peru | Zinc | 33,02 |
