# Supplementary material for: The impact of different agroecological conditions on the nutritional composition of quinoa seeds
Source: PeerJ. 2018 Mar 14;6:e4442. doi: 10.7717/peerj.4442 (PMC5857176; doi:10.7717/peerj.4442)
Supplement: Data S6 — Phytate was determined in seeds according to the methodology described in Methods. Final data is presented in Fig. 2. [file peerj-06-4442-s008.docx]

| **VAR LOC** | **% Phytate g/100g** | | **1** | **2** | **3** | **4** |
| --- | --- | --- | --- | --- | --- | --- |
| **Salcedo Spain** | 0.318 ± | 0.054 | - | 0.370 | 0.263 | 0.322 |
| **Regalona Spain** | 0.398 ± | 0.027 | 0.389 | - | 0.376 | 0.429 |
| **Titicaca Spain** | 0.329 ± | 0.091 | 0.451 | 0.341 | 0.241 | 0.284 |
| **Salcedo Chile** | 0.271 ± | 0.054 | 0.307 | 0.298 | 0.191 | 0.288 |
| **Regalona Chile** | 0.239 ± | 0.056 | - | 0.243 | 0.181 | 0.292 |
| **Titicaca Chile** | 0.198 ± | 0.029 | - | - | 0.177 | 0.219 |
| **Salcedo Perú** | 0.239 ± | 0.058 | 0.203 | 0.211 | 0.217 | 0.325 |
